# Molecular Biophysics Database (MBDB) makes raw measurements findable and reusable

**DOI:** 10.1007/s00249-025-01789-1

**Published:** 2025-08-10

**Authors:** Emil Dandanell Agerschou, Terezie Prchalová, Miroslav Šimek, Michal Malý, Jan Stránský, Michal Strnad, Andrea Santisteban-Veiga, Mark A. Williams, Juan Sabín, Jan Dohnálek

**Affiliations:** 1 Institute of Biotechnology of the Czech Academy of Sciences, Průmyslová 595, 252 50 Vestec, Czech Republic; 2 Czech Education and Scientific NETwork (CESNET), Generála Píky 430/26, 160 00 Prague 6, Czech Republic; 3 AFFINImeter, Software 4 Science Developments, Edificio Emprendia, Campus Vida, 15782 Santiago de Compostela, Spain; 4 Institute of Structural and Molecular Biology, School of Natural Sciences, Birkbeck, University of London, Malet Street, London, WC1E 7HX UK

**Keywords:** Molecular biophysics, Database, Repository, Raw data, Metadata model, FAIR principles

## Abstract

Open science is now established as an important paradigm for publicly funded research. The main principle being that to ensure best use of research data and integrity of the scientific process the information from experiments should be made widely and freely available. However, dedicated technical infrastructure to enable useful access to comprehensive experimental information in molecular biophysics is lacking, in particular in regard to repositories for raw measurement data. The Molecular Biophysics Database (MBDB) was created to fill this gap. The MBDB provides a common and extensible framework to store and access raw measurement data from a growing number of biophysical methods, currently including bio-layer interferometry, isothermal titration calorimetry, surface plasmon resonance, and microscale thermophoresis, with additional methods planned for the future. Alongside the raw measurement data from these methods, a rich set of metadata to enable data reuse is captured in accordance with the FAIR data management principles. An overview of the data models and technologies that were used to create the MBDB is presented here.

**Supplementary Information:**

The online version contains supplementary material available at 10.1007/s00249-025-01789-1.

## Introduction

**Fig. 1:**
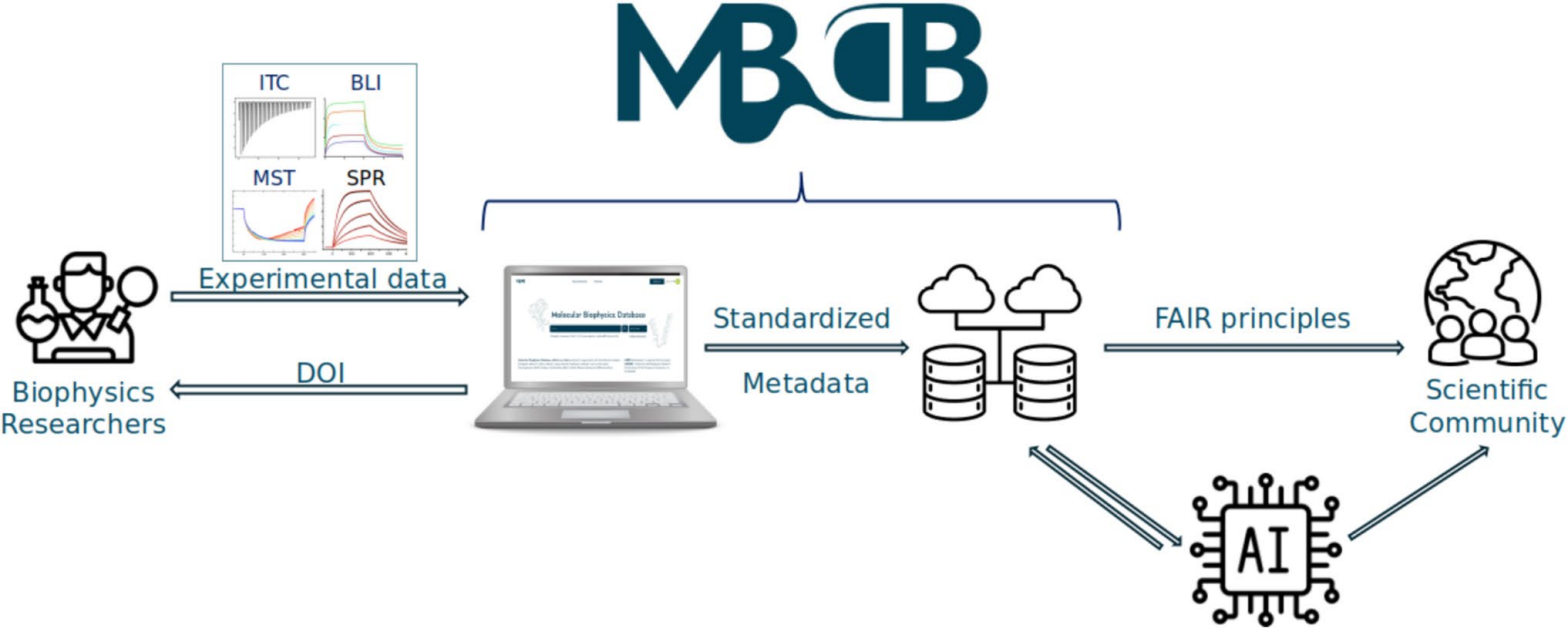
Overview of the role of the MBDB in mediating the interaction of the researcher with the scientific community. The MBDB captures experimental data and metadata in a standardized format and stores and provides the data to the scientific community for potential verification and reuse, including the possibility for large-scale analysis by AI and other computational methods

Science strives for openness regarding how any given piece of knowledge is generated. Sharing evidence enables verification or refutation and builds trust in the process. Openness, however, is not only a moral imperative, but also a way to acknowledge that we could have inadvertently made an error or used an implicit or incorrect assumption in reaching our conclusions. If the creators of data are the only ones to ever see it, such errors in our collective knowledge are hard to discern and correct. Furthermore, obtaining experimental data requires a great deal of expertise, resources, and time. Given this significant investment, it is surprising that the generated data in the molecular biophysics is commonly treated as single-use only and not usually considered worth preserving in a durable and public way. Consider the following scenarios: (1) a researcher who normally measures protein–protein interactions finds herself measuring protein-vesicle interactions. The researcher observes an artefact related to vesicles and wants to investigate if it has been seen before. (2) A researcher has developed a new method for data analysis and wants to assess how this reanalysis would alter published results. (3) A researcher wishes to compare and combine their measurement of a particular biomolecular reaction with measurements made in other laboratories. In each case, literature research and lengthy email correspondence would be necessary to obtain the needed raw measurement data, even if it was still extant. Had the raw data been published according to the FAIR (Findability, Accessibility, Interoperability, and Reusability) principles (Wilkinson et al. 2016; Jacobsen et al. 2020), access would have been straightforward. The newly developed Molecular Biophysics Database (MBDB) aims to achieve just that: Making raw data from molecular biophysical experiments available, (re)interpretable, and citable (Fig. 1).

Before addressing specific data items in the MBDB, it is worth defining the different types of information that can be captured in general.

## Data

The MBDB uses the following data categories: Raw measurement data—numerical data directly obtained from instruments (e.g., fluorescence intensity over time, stack of images), which are stored in their original file format. Derived measurement data—raw measurement data that has been processed (e.g., background-subtracted data, reduced to counts of objects with specific intensity), also stored as separate files. Results—biophysical parameters derived from the data that characterise molecular processes (dissociation constants, stoichiometries, etc.), which are embedded with their metadata rather than stored in stand-alone files.

## Metadata

Metadata provide the context within which the data should be understood. All three types of data mentioned above have associated metadata. Broadly, the metadata serve to fully describe the molecular system being studied [including sequences, UniProt identifiers (The UniProt Consortium 2025), small molecule ligands and buffers, InChI keys (Heller et al. 2015), etc.], to explain who generated the data (e.g., people, affiliations, and grants), how it was generated (instrument settings, pH values, data normalization, etc.), and how it was analysed to produce biophysical parameters.

## Data model

The data model is the structure of the captured metadata and data. Conceptually, the data model can be thought of as a spreadsheet with named columns and each record can be thought of as a row of values. A central concept in this structure is the field (named column). A field in the context of MBDB is a named element with a description, a data type, and possibly a constraint. It is important to note that the data type of a field can be rather complex and may include several layers of nested fields (spreadsheets within spreadsheets).

## Accessibility of raw measurement data in molecular biophysics

With the exception of methods providing information on the three-dimensional structure of molecules, efforts to collect and present molecular biophysics data (raw or derived) in a standardized and reusable format have been limited. In our view, this was caused by four main factors: (1) the large variety of techniques being applied in the field, which utilize sophisticated observation of diverse physical parameters under defined conditions (e.g. spectra and other optical properties, heat and other thermal properties, hydrodynamic properties); (2) lower perceived need by the research community compared to structural molecular data; (3) difficulties in agreeing on the definition of metadata necessary to find and reinterpret raw data, with no universal standard until now; (4) variability (and opacity) of the output file formats from instruments being used to make measurements.

One approach to overcoming this lack of provision might be to follow the current practice of neighbouring disciplines and establish separate standards and databases dedicated to each method. This has the virtue of tailored design and storage provision for specific types of data, but the disadvantage of adding complexity to the landscape of open science. For example, in structural biology, a pioneer of open science, standard formats for storing interpreted results (structures) and metadata have long been established. Currently, mmCIF (Bourne et al. 1997) serves as the de facto standard for X-ray crystallography, NMR, and cryo-EM structure determination experiments. However, because of both the distinct nature of these methods and their different historical development, underneath the standard, even basic information such as the macromolecular chemical environments is recorded in separate contexts i.e. there are separate standards for crystal growth conditions, NMR sample details and cryo-EM buffers. Furthermore, when raw and derived experimental data are available, each method has separate databases dedicated to data storage (The wwPDB Consortium 2024; Morin et al. 2013; Grabowski et al. 2016; Hoch et al. 2023).

### Databases for raw molecular biophysics data

As for non-structural molecular biophysics techniques efforts have been made to record Circular Dichroism (CD) measurements (PCDDB, Whitmore et al. 2011 and NACDDB, Cappannini et al. 2023) and mass spectrometry/proteomics data (PRIDE—the PRoteomics IDEntifications Archive, Perez-Riverol et al. 2025). The Protein–Ligand Binding Database (PLBD, https://plbd.org, Lingė et al. 2023) provides considerable depth of information on a small number of experimental systems measured using four different experimental methods and aiming to guide understanding of the structure–activity relationships. However, it is not aimed at becoming a general repository of raw data for one or more biophysical techniques.

For circular dichroism spectroscopy (a technique with both structural and non-structural application) a separation has occurred by biomolecular system being studied with the PCDDB and NACDDB capturing CD spectra and metadata from proteins and nucleotides, respectively. It is consequently unclear where spectra from samples containing both types of macromolecule should be deposited.

To the best of our knowledge no other techniques have been covered. Recently, we have performed a detailed survey of the currently available databases in the field of biomolecular research (Stránský et al. 2022). Very few resources provide FAIR options for raw data deposition under generally acceptable standards.

## Concept of Molecular Biophysics Database

Given the present situation of limited provision for non-structural molecular biophysics there is the possibility of avoiding this fragmentation from the outset. The MBDB aims to provide a single framework that can accommodate data from many methods. This aim is greatly assisted by the fact that, although there are many methods based on different physical principles, they are typically being applied to derive the same parameter (dissociation constant, on-rate, stoichiometry, etc.), that methods are often applied in similar ways (investigating a change to a biomolecular behaviour as a result of varying concentration, temperature or with time) and that they are similarly sensitive to experimental sample content and thus have similar requirements for detailed description of the sample. Consequently, there is a considerable commonality of necessary data elements. Furthermore, many researchers use multiple biophysical methods and a single database makes more sense from a perspective of encouraging adoption and sustainability of the resource.

Some of the very few other public resources for storage of non-structural biophysical raw data are in the field of mass spectrometry (MS). The ProteomeXchange consortium comprises several MS proteomic databases which have worked together to create MS data exchange formats and set a common core of metadata required for deposition to enable findability and interoperability of these data. The largest of these databases, PRIDE is a repository for mass spectrometry proteomics data, including protein and peptide identification, the corresponding expression values, post-translational modifications and the raw mass spectra (Perez-Riverol et al. 2025) often with rich experimental details and other metadata. However, the experimental data are coming from complex systems (such as cell lines and tissues). Much of the metadata describing these systems has not been standardised and is stored using a free text format, making it difficult to identify equivalent or comparable datasets.

In contrast, many molecular biophysics experiments are carried out on well-defined systems and by diverse, but also clearly specified, methods. Standardisation in molecular biophysics is, thus, most useful regarding the definition of metadata elements and the format(s) for expressing them, to maximise the opportunity for data reuse, reanalysis, and comparison across different biophysical methods. Consequently, the MBDB takes the approach of standardising the metadata, and preserving the raw data in its original (non-standard) format, which is sufficient to make the data concerning specific biological systems findable, accessible, and reusable in principle.

Standardisation of raw measurement data in molecular biophysics is not an achievable goal in the near-term. The diversity of file formats used across different methods, even within individual methods, multiple instrument manufacturers and software versions create very many format and content variations. Current data storage and transport formats in molecular biophysics include flavours of XML, HDF-5, SQLite, XLSX, variations of TSV/CSV, and proprietary formats. In the most difficult cases, raw experimental data files are only presently reusable by a subset of the scientific community that have an appropriate manufacturer’s software licence. The MBDB’s role where data are not yet freely reusable is to preserve data for the possibility of future reanalysis, e.g., by third party software such as the eSPC suite of tools (Burastero et al. 2021).

Of immediate concern to the MBDB is to specify the experimental system under investigation and the experimental protocol as precisely and clearly as possible. The available metadata describing the experimental setup in the files containing the raw measurements coming from different instruments vary greatly in detail and frequently appear to have been designed to be used only by the measurement/data analysis software associated with a particular instrument. Such files tend to use specific terms rather than standard terms or ontologies for the method and to have difficult to read formats. To solve this problem for each method a standardised common subset of data necessary to evaluate the results has been developed in consultation with the scientific community.

The work presented here focuses on the implementation of the Molecular Biophysics Database, the common metadata standards for all methods and the additional metadata required for each method that have been implemented to date.

## Implementation

The MBDB application is implemented on top of the robust InvenioRDM framework (https://inveniordm.docs.cern.ch/), a scalable solution designed for creating digital repositories. InvenioRDM is widely deployed and ensures long-term stability for future development. Of note, Zenodo, the major general-purpose, open-access repository operated by CERN in collaboration with OpenAIRE (https://www.openaire.eu/) is an InvenioRDM-based system. InvenioRDM is developed using Flask, a popular Python micro-framework that facilitates rapid development and supports a modular architecture. This design enables efficient handling of metadata, versioning, and access control for digital content.

In the MBDB application, a core InvenioRDM system is augmented by additional functionality provided by the OARepo project (https://github.com/oarepo), a key component of the Czech National Repository Platform. This combination leverages the robustness, scalability, and API accessibility of the InvenioRDM platform with custom metadata schemas specifically developed for molecular biophysics experiments. To support these features, a customized user interface for data deposition, search, and access has been developed.

Internally, the metadata model in InvenioRDM-based digital repositories relies on JSON, an ECMA-404 standard, with metadata stored in a PostgreSQL database. Indexing is performed using OpenSearch, while metadata validation is executed with a Marshmallow schema at the business-logic layer and further verified by a JSON schema prior to persistence.

Data files are stored redundantly using S3 buckets on Ceph (https://ceph.io/en/), ensuring high storage reliability and availability. In addition to managing data and metadata, persistent and citable global identifiers, notably DOIs, are minted via API calls to DataCite, with metadata converted to DataCite’s schema (DataCite Metadata Working Group 2024).

User authentication is managed through ORCID (https://orcid.org/) using the OAuth protocol.

All the above-mentioned technologies are open source, including the MBDB itself (see Data Availability section).

## Overview of the (meta)data model

In designing the (meta)data model, three conflicting goals were balanced: i) to enable uniform searching across methods, ii) to capture the specific details needed to reproduce or (re)evaluate a dataset, and iii) the ability to extend the model to capture new methods.

**Fig. 2:**
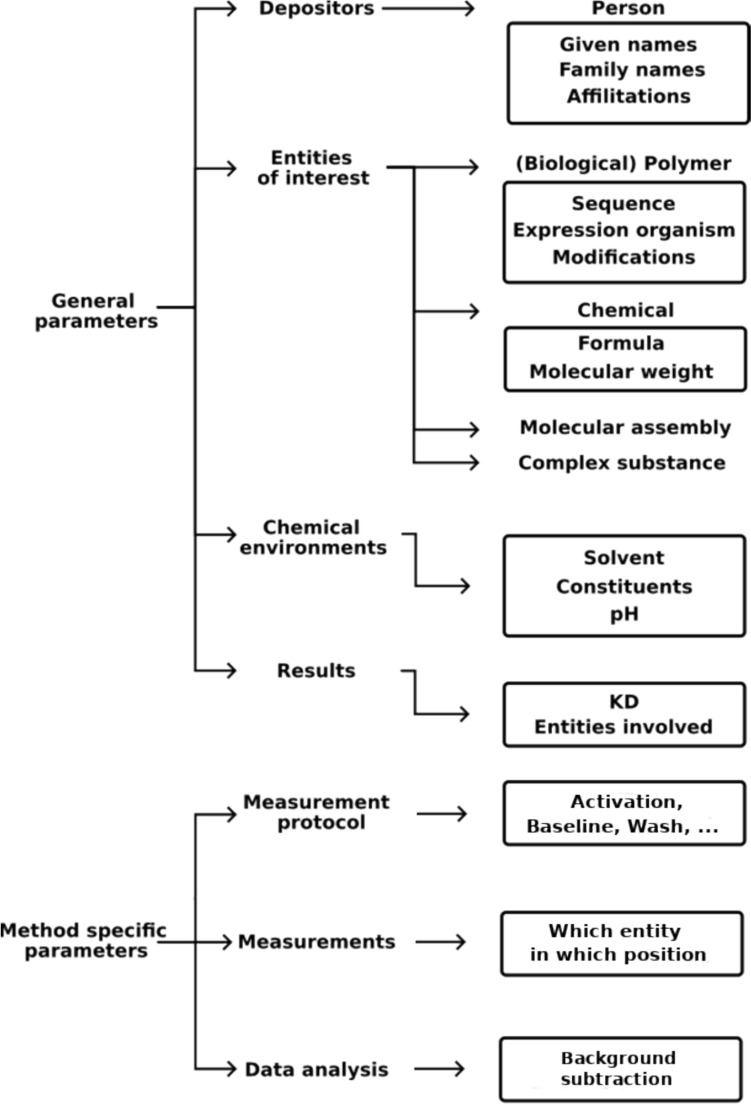
Schematic overview of selected metadata required upon deposition of a raw data file to the MBDB. The requirements are closely tied to the underlying data model. The high-level categories of information in the central column are described in the main text. The boxes list a few examples of the more than three hundred possible data elements. Schemas of selected metadata categories are provided in the Supplementary material

A balance was struck with a two-part model (Fig. 2). The first part is a set of general parameters that are shared among all methods, and the second part is specific to each method. Among the general parameters are bibliographic information, identification of measured molecular entities, details of chemical environments, and the results of measurement. Among the method-specific parameters are instrument settings, measurement protocols, quantities describing individual measurements, and data analysis as these can vary dramatically between different methods. Currently, the number of data fields of the general part is significantly higher than of those in the method-specific part (a complete record may be formed by 5–20% of the method specific parameters, depending on method and the depositor’s approach). The alternative approach of a model general enough to capture all variations under one roof would be very difficult to understand. Note that this in practice means that while the use of general parameters minimises duplication, there are as many second parts to data models as supported methods. This separation of method specific parameters makes it possible to add new methods without any risk to existing records.

A guiding principle in the creation of the data model was to reuse elements as frequently as possible to ensure uniformity. An example of this is the chemical notion of a concentration. Excluding results, in all other parts of the model, concentration has the same layout (value, unit), which allows for uniform searching. This also greatly simplifies the data model, allowing it to be efficiently maintained by a small number of people. Another important aspect of the data model is that, similar to mmCIF, the description of each item is embedded within the data model itself. This ensures that the semantic meaning of the fields never have to be guessed. Furthermore, embedding the description within the model allows tracking the changes to the descriptions as these changes might affect what users input into the field.

## Vocabularies

Certain data elements are annotated using so-called vocabularies. Vocabularies play a similar role in the InvenioRDM as foreign keys do in relational databases. They are, in simplified terms, a very useful mechanism to annotate objects that are likely to be used in multiple records. An example of this is basic chemical information for a particular chemical compound pulled from the PUG-REST of PubChem (Kim et al 2018, 2025). Unifying basic chemical information improves searchability, internal consistency, and makes it easier to find further information about any compound in external databases as external IDs are stored and updated. Vocabularies using external identifiers include the Research Organization Registry (ROR) for affiliations, OpenAIRE Projects for grants, and the NCBI taxonomy for biological species (Schoch et al. 2020). Furthermore, vocabularies that are internal to the MBDB are also present, including for instance names of measurement instruments.

## Overview of general parameters

### Entities of interest

The “entities of interest” field is crucial to make the records findable in the MBDB. This is the metadata object that captures information about the entities that are being studied (macromolecules, chemicals, biological fluids, etc.), as well as the entities that are being employed to modify their behaviour. Given the diverse range of experimental samples that can be subject to biophysical analysis, the entities fall in two broad categories i) those that are chemically well-defined at the time of the measurement (polymers, chemicals, and macromolecular assemblies) and, ii) substances that are chemically complex, but well-defined by the protocol used to collect them, which are grouped by their origin (biological, environmental, industrial, or chemical). For the chemically well-defined entities, metadata is focused on the molecular details, while for the complex substances, the focus is on the preparation and its composition (Fig. S1 of the Supplement).

Because the results of a biophysical measurement (e.g., a dissociation constant for a protein-small molecule interaction) are sensitive to the precise chemical nature of the reactants, the data model for chemical entities is very detailed and customisable. Where possible, named entities are derived from existing external databases and have standard representations. This is the case for small molecules where InchI Keys (Heller et al. 2015) are used as unique identifiers and data concerning the molecular entity are drawn from PubChem (Kim et al. 2025) where they exist. For custom small molecules, such as might be created in a pharmaceutical context, there is the option to add user-defined molecular data. In the case of macromolecules, links can be made to external data sources, e.g., Uniprot for proteins, which make additional contextual information available. However, the principal definition of the macromolecule (polymer) is its sequence of amino-acids or nucleotides, including provision for annotation of post-translational or chemical modification. This is because the results of measurements will be dependent on the precise chemical nature of the macromolecule and its sequence will not usually correspond to the gene sequence, but to a fragment thereof that may be tagged or mutated. Polymer sequences also allow for the inclusion of novel synthetic molecules not related to any gene. Each defined sequence is associated with a unique identifier in the database such that it can be reused in multiple entries. Information concerning the evidence for purity of chemical entities can also be included.

### Chemical environment

Molecular biophysics measurements of, for example, the formation of a complex of a macromolecule and small molecule ligand, take place in a surrounding chemical environment (e.g., comprising the solvent, buffers, NaCl, glycerol, DMSO, urea, etc.) that very often will influence the measurement results. Consequently, the environment must be precisely defined in terms of its constituents (each of which is a defined chemical entity) and their concentrations. Furthermore, capturing the constituents in a structured fashion makes them searchable, hence records can be found based on constituents. The Chemical environment metadata of the MBDB are illustrated in a scheme shown in Fig. S2 of the Supplement.

### Results

Results within the MBDB are defined as the physical parameter that is sought by the experiment (e.g., a dissociation constant). A result will correspond to one or more of the specified entities of interest present in the record. Results are useful as evidence of the authors interpretation of the experimental data, for searching, and for making clear the link between values in any article publication and the deposited experimental data. However, as the focus of the MBDB is on the raw data, results are optional.

## Overview of method specific parameters

As biophysical methods vary widely, so do their associated method specific parameters. However, they generally include information related to the instrument (model and manufacturer), the instrument settings during the measurement (including temperature), and other aspects of the measurement protocol, and the final composition of the samples (entities of interest, their concentrations and the chemical environment) that were measured, and which correspond to a raw measurement file. The data analysis section includes the equilibrium or kinetic model used to analyse the data to obtain results including, for example, information on the analysis software used. Selected parts of the method specific metadata of the MBDB are illustrated in schemas in Figs. S2–S6 of the Supplement.

## Front-end design

**Fig. 3:**
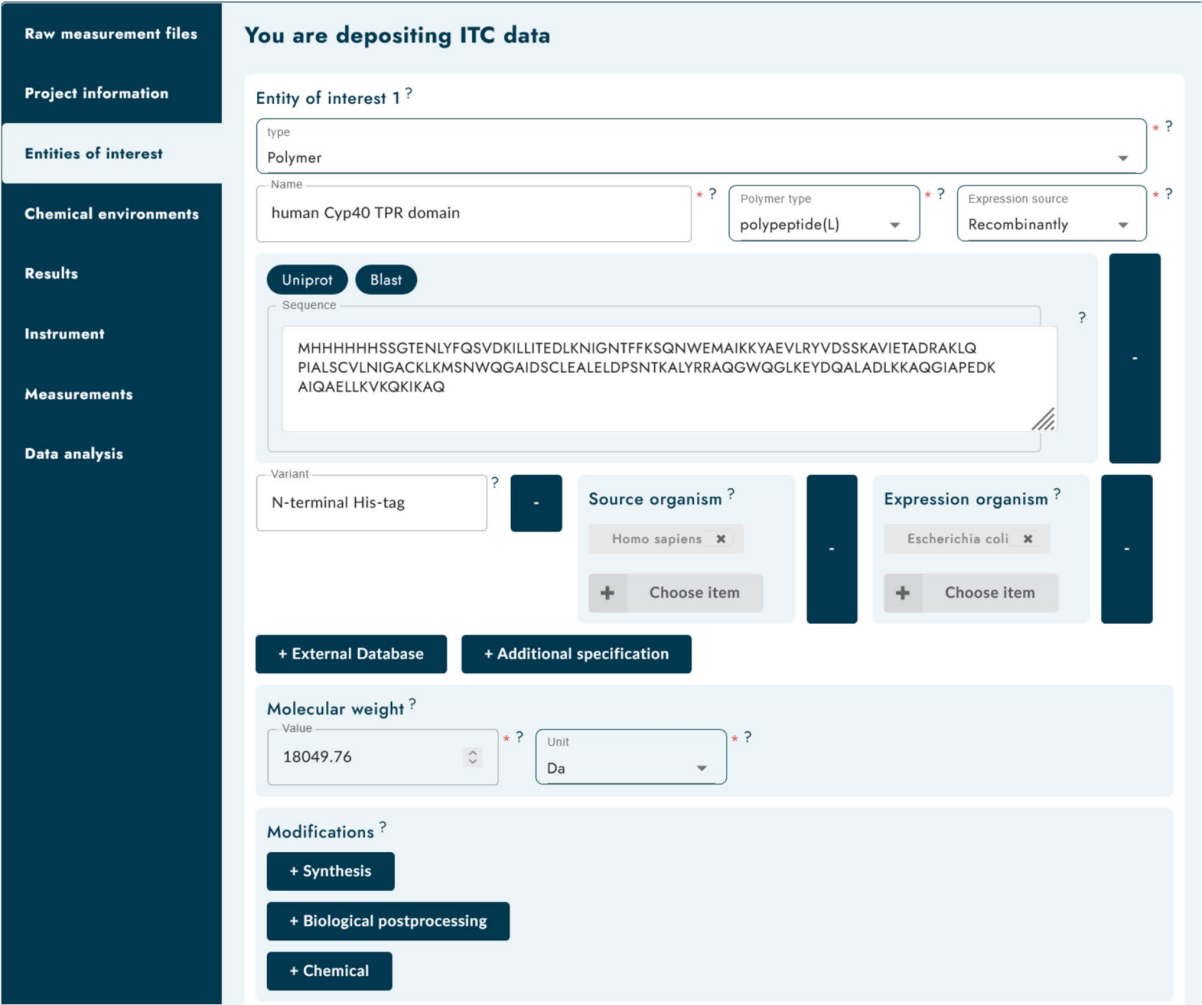
Screenshot from the MBDB user interface for a record showing the partial exposure of fields for the entry of *Polymer* type of Entity of interest

The frontend, in particular with respect to the deposition form, is centred around making it as easy as possible for the depositors to fill in the required information. This requires a responsive and dynamic user interface (Fig. 3), which is implemented using the React framework (https://react.dev/). Tabs are used to structure the data input task. Each tab corresponds to a category of information in Fig. 2 and collects required and optional data values for that category. The dynamic interface means that for each tab only a small number of fields are initially visible to the user, and as the user enters data this number increases in response to data values entered. For example, for the chemical entities tab, first a type of chemical entity is requested, if *Polymer* is chosen, then a sequence box appears into which the sequence can be pasted, together with other relevant fields for polymers, including polymer source and expression organisms, and types of modification (Fig. 3). If a biological postprocessing modification is then selected, additional fields appear to enter further details. This progressive exposure of fields reduces the risk of the user getting overwhelmed during deposition, i.e., by only exposing necessary fields and not the entire data model.

The frontend user interface is divorced from the data model itself, and this enables continuous development to further improve the deposition experience. However, divorcing the frontend from the data model comes with some drawbacks. In particular, it makes it less intuitive for the user to search through records as they are not aware of the underlying structure of the record, and it requires additional work to synchronise the frontend with any change to the data model. Development experience and user testing have shown that the advantages of a flexible interface outweigh the drawbacks.

## Supported methods

Implementation of methods was prioritised based on a previous survey of needs for databases and data standardization among biophysics researchers (Dohnálek et al. 2022). Criteria for prioritising methods were: Lack of existing data repository for the method, high usage and number of users, and the perceived need for standardisation by the survey participants. This led us to choose the following currently supported methods:

• Bio-layer interferometry (BLI)
• Isothermal titration calorimetry (ITC)
• Microscale thermophoresis (MST) and related Nanotemper methods including the 'spectral shift' and 'initial fluorescence'
• Surface plasmon resonance (SPR)

Although deposition is currently limited to the four above-mentioned methods, further methods will be incorporated into the MBDB. The eligibility criteria for new methods are similar to the initial inclusion criteria, namely:

• It should be within the scope of the field of molecular biophysics
• It should not have an existing standard deposition resource for raw data
• It should have an established user community
• The community should be interested in co-developing the standards and participate in peer-review of the records

Currently, Mass photometry is the next planned method for the creation of a metadata model, and other methods are being evaluated for inclusion, namely DSF, FRET, and 1D NMR.

## Publication workflow

**Fig. 4:**
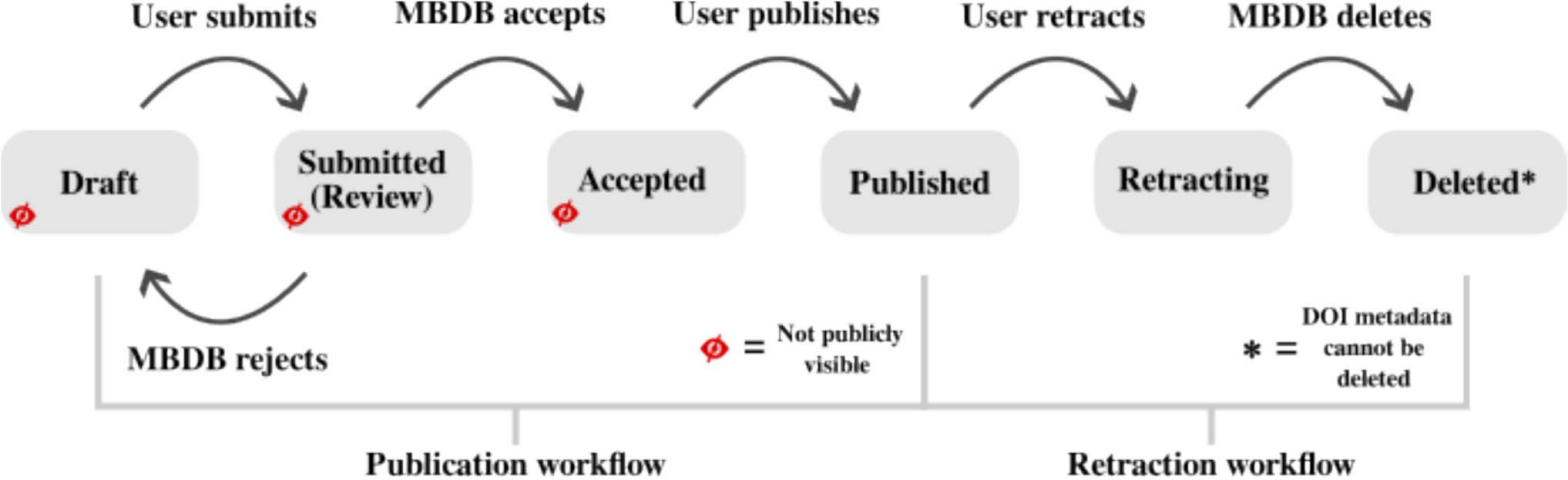
Schematic overview of the publication and retraction workflows for a record deposited in the MBDB

Making depositions to the MBDB follows a very similar route to that of scientific articles published in peer-reviewed journals (Fig. 4). The User creates an account on the MBDB (the only requirement is an active ORCID account); at this point the user can initiate a record and create a draft using the deposition form. Drafts are not publicly visible. When the user is satisfied with their record, they can submit it for review. MBDB validates the record and in case there is an error, the record is rejected with information about why. It is important to note that during review, the MBDB is not judging the science of the record, merely whether the information is complete or shows signs of mistakes, contradictions, or evident fraud. If/when the record is accepted, it is not published immediately. The user is free to publish the record whenever they choose to do so, at which point it becomes public and a DOI is generated. The record is then distributed under the Creative Commons license (https://creativecommons.org/publicdomain/zero/1.0/legalcode), which means that it is placed in the public domain. If the user decides to retract the record, they can do so and the MBDB would stop distributing the data. However, part of the metadata associated with the record (the metadata needed for the DOI) cannot be fully deleted as the record might have already been cited, so people who use the DOI need to know that they found the record but that the data is no longer available. Furthermore, as the data was placed in the public domain, existing copies of the data can be legally distributed by MBDB users who already acquired them.

## Overview of user interaction with MBDB

The user’s interaction with the MBDB is currently limited to basic search, basic inspection of records and download of both data and metadata.

The search is implemented using the OpenSearch technology. The user can select whether the search is performed against all records, or specific to a biophysical method. To improve the performance, only a limited set of record items are indexed; the subsets differ for the full search and the method-specific search.

A simple view of the individual record is available in the user interface (UI); only a subset of parameters is visualized to lessen the visual overload of the user. The complete set of metadata parameters is available for download in JSON format, as well as the original measurement data files in formats as provided by depositors. The download is accessible in the simplified record view.

Programmatic access to the database is possible using REST API (Representational State Transfer Application Programming Interface). Published records can be retrieved without authentication, however, data deposition and retrieval of record drafts is possible only with the token-based authentication. The personal API tokens can be retrieved using the web UI upon user login.

A detailed record view and advanced search are currently under development.

## Discussion and future developments

The focus on findability and uniform searching at first glance might appear to primarily benefit researchers that use the data rather than the researcher who spends the effort to deposit the data. However, the most likely users of the data are the depositors themselves. One of the benefits of deposition is that depositors have a way to safely store, find, and compare their own data. Well annotated data stored in a public repository are no longer at risk of physical loss from local computer failures or loss of interpretability from staff turnover. Furthermore, obtaining a DOI via DataCite makes the data citable, both directly by other researchers but also by the depositors themselves to prove their research output. This also largely improves the situation of research infrastructures providing measurement services and being requested by funders to show evidence of measured data sets.

The MBDB not only makes it easier for individual researchers, but it also makes it easier for the communities around each method. In particular, it makes it easier to compare measurement protocols across different depositors in a systematic way to find ways to optimise or create consensus measurement protocols. Secondly, it gives the communities a focus to discuss further development of metadata standards and data validation.

Collecting data in a structured form allows for systematic reuse and data mining in the future. This is both true for those who wish to apply various machine learning techniques, improve measurement protocols, or improve instrumentation and data analysis tools.

A comparison of MBDB with PCDDB, NACDDB, PLBD, and PRIDE shows the diverse focus of the individual resources. While MBDB functions as a repository open to curated depositions of researchers’ data sets, PCDDB is focused only on reference quality datasets for protein CD and has no direct deposition option. With MBDB the quality of deposited data is the main responsibility of the depositor. NACDDB is populated by the database authors with very limited metadata regarding the actual experimental conditions and samples; it does not enable direct data deposition. PLBD provides an interface to interpreted data and results of protein–ligand interaction measurements with some metadata regarding the measurement conditions but the underlying raw data are not accessible and direct data deposition is not the purpose of this resource. PRIDE accepts depositions of raw mass spectrometry data and to some extent enables recording of experimental metadata in a free text format. MBDB strives to adhere to the FAIR rules of data management by systematically capturing experimental conditions critical for the outcome of any biophysical measurement and making data searchable, findable and accessible for reference or for reuse. The minimally required metadata parameters are designed in such a way that it should be possible to download an MBDB record and re-process it, given the necessary software is available. Furthermore, the minimal required metadata should be sufficient for repeated experiments under the same conditions, depending only on the availability of samples and similar instrumentation.

Future developments to improve the user interface to make it easier to deposit and retrieve data will continue. This will include further development of the API, which provides a route for infrastructures and other large producers of data to create their own automated deposition tools. Users who wish to develop such tools and publish their data via the API can contact the MBDB to obtain the necessary credentials.

Direct linking of records to external databases and resources for biomolecular research is planned to enable easier data interpretation and identification of related data records of other type than those in the MBDB.

Lastly, the MBDB is developing automation of data input/extraction from raw data formats for the most used methods and instruments to make it easier for the user to reuse the data but also to enable better validation at the point of record review.

## Supplementary Information

Below is the link to the electronic supplementary material.

Supplementary file1 (PDF 1499 kb)

## Data Availability

The deposition and searching of data can be done at https://mbdb-data.org which is the official website of the MBDB. The MBDB code base is open source under an MIT license and hosted on GitHub https://github.com/Molecular-Biophysics-Database where bug-fixes and other issues can be raised. We welcome contributions to development.
