# Supplementary material for: Molecular Biophysics Database (MBDB) makes raw measurements findable and reusable
Source: Eur Biophys J. 2025 Aug 10;55(3):335–45. doi: 10.1007/s00249-025-01789-1 (PMC13319166; doi:10.1007/s00249-025-01789-1)
Supplement: Supplementary file 1 — Supplementary file1 (PDF 1499 kb) [file 249_2025_1789_MOESM1_ESM.pdf]

Supplementary information
to
Molecular Biophysics Database (MBDB) makes raw measurements findable and reusable

Emil Dandanell Agerschou[1], Terezie Prchalová[1], Miroslav Šimek[2], Michal Malý[1], Jan Stránský[1], Michal Strnad[1,2], Andrea Santisteban-Veiga[3], Mark A. Williams[4], Juan Sabín[3], and Jan Dohnálek[1]

(1) Institute of biotechnology of the Czech academy of Sciences, Průmyslova 595, 252 50 Vestec, Czech Republic
(2) Czech Education and Scientific NETwork (CESNET), Generála Píky 430/26, 160 00 Praha 6, Czech Republic
(3) AFFINImeter, Software 4 Science Developments, Edificio Emprendia, Campus Vida, 15782, Santiago de Compostela, Spain
(4) Institute of Structural and Molecular Biology, School of Natural Sciences, Birkbeck, University of London, Malet Street, London WC1E 7HX, United Kingdom

Metadata structure

General metadata

Only the upper layers of selected data model blocks are illustrated here. For more details, please see https://github.com/Molecular-Biophysics-Database, especially the data model definitions https://github.com/Molecular-Biophysics-Database/mbdb-model.
The metadata structure is shown here as organized in the UI of the MBDB.

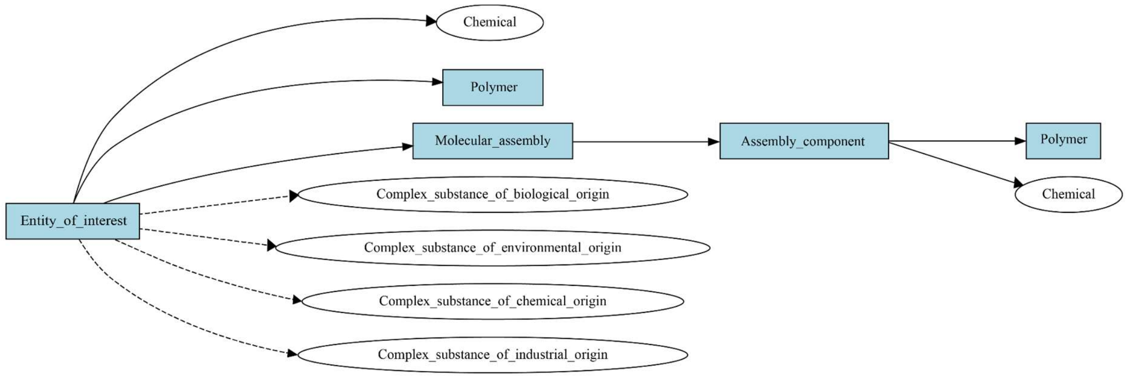

Fig. S1 Schema of the "Entities of interest" following the logic of data organization and hierarchy (omitting technical details) of the MBDB general metadata. For details see the full data model definitions.

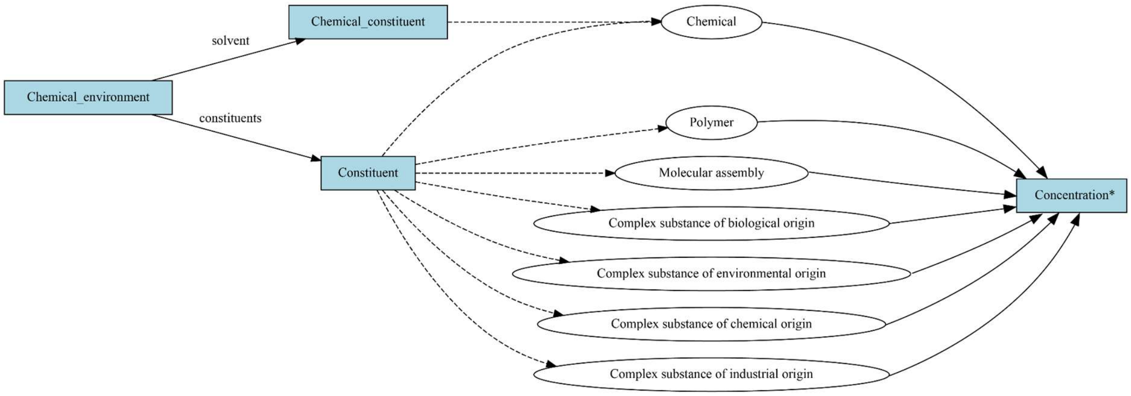

Fig. S2 Schema of the “Chemical environment” following the logic of data organization and hierarchy (omitting technical details) of the MBDB general metadata. * Concentration is given as an example of a required field; parameters for the individual types differ. For details see the full data model definitions.

Method specific metadata

Microscale thermophoresis

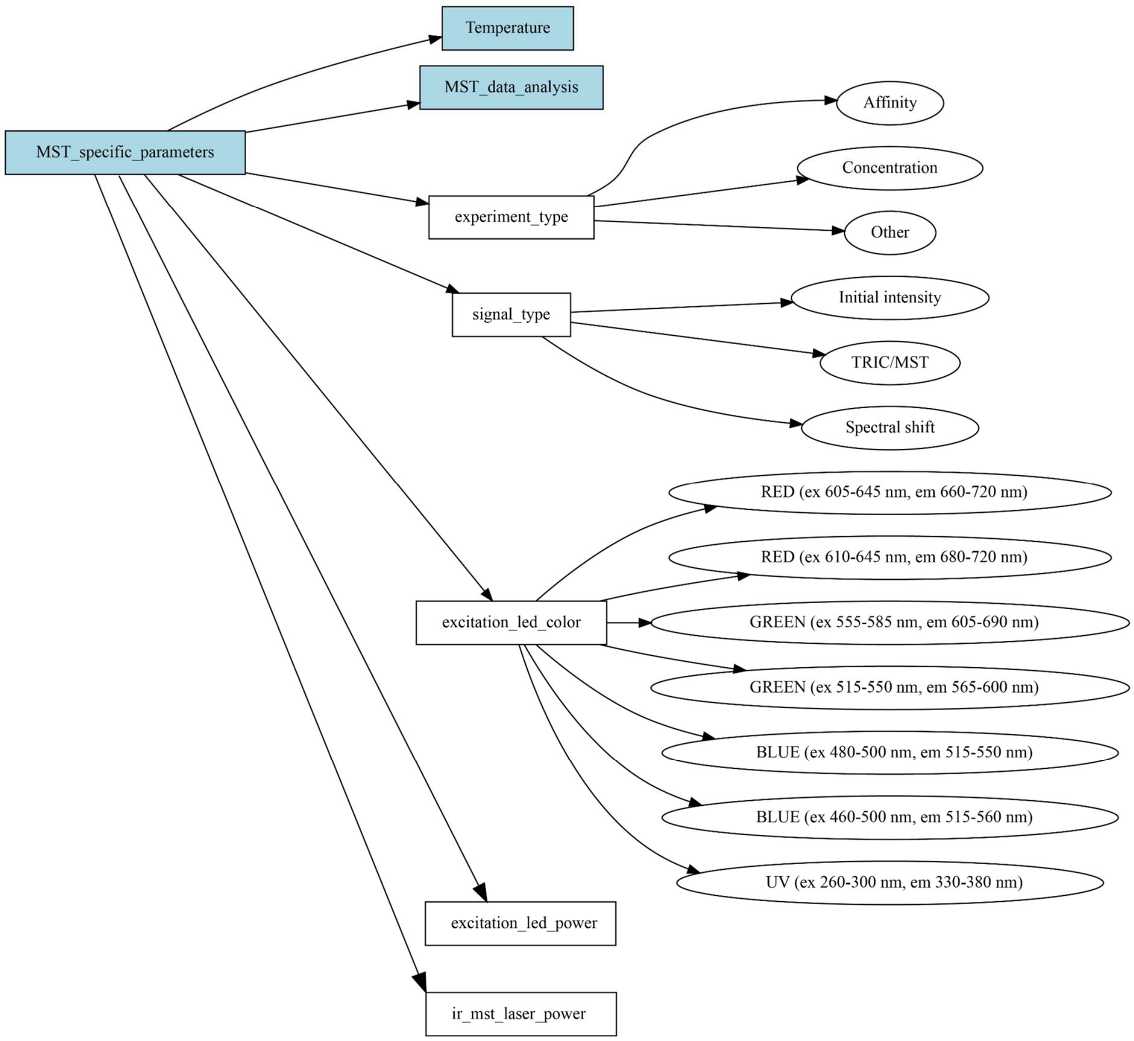

Fig. S3 Schema of the MST-specific metadata related to method type and instrument settings, under the data block “Instrument” of the MBDB UI. For details see the full data model definitions.

Bio-layer interferometry

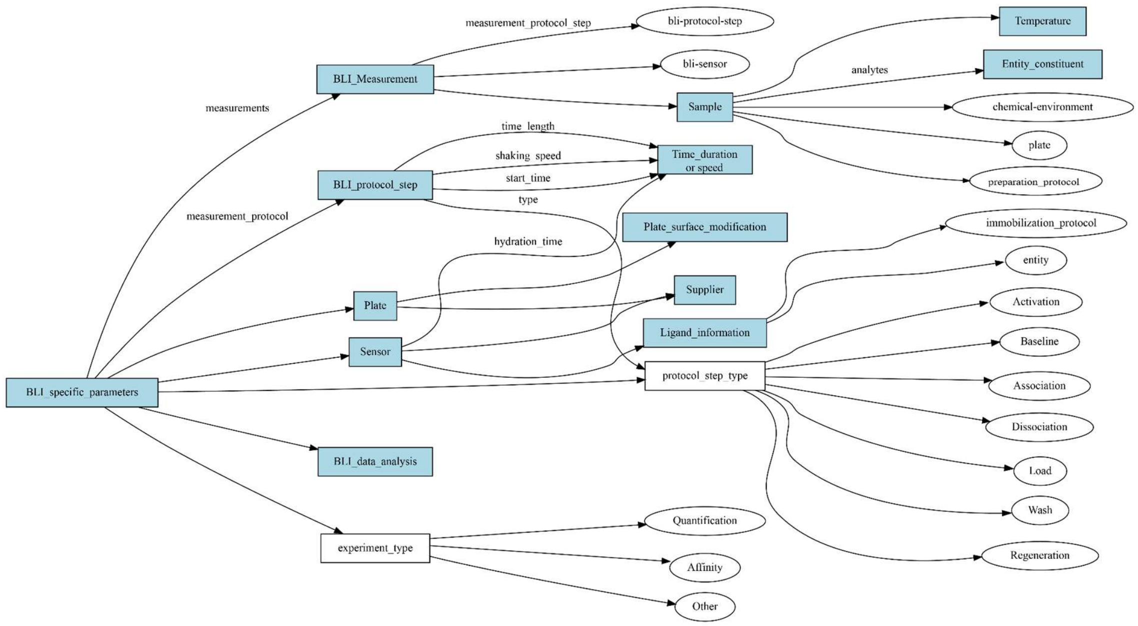

Fig. S4 Schema of the BLI-specific metadata related to instrument settings, measurement conditions and protocol, under data blocks “Instrument”, “Plates”, “Sensors”, “Measurement protocol”, and “Measurements” of the MBDB UI. For details see the full data model definitions.

Surface plasmon resonance

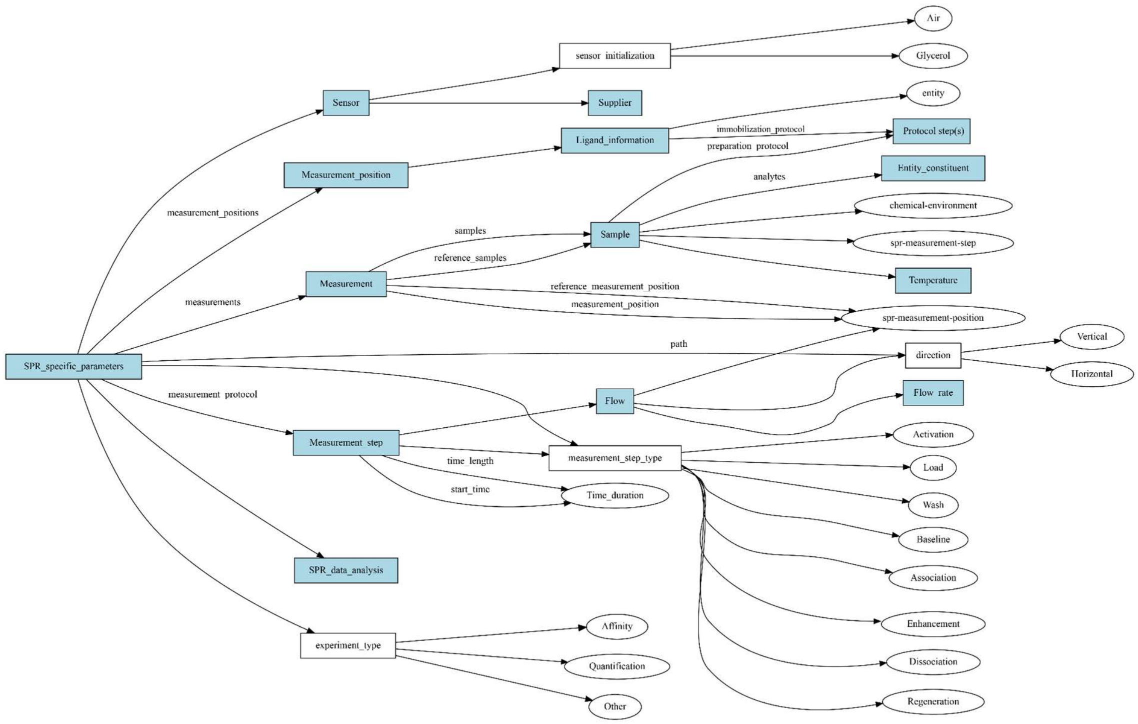

Fig. S5 Schema of the SPR-specific metadata related to instrument settings, measurement conditions and protocol, under data blocks “Instrument”, “Sensor”, “Measurement positions”, “Measurement protocol”, and “Measurements” of the MBDB UI. For details see the full data model definitions.

Isothermal titration calorimetry

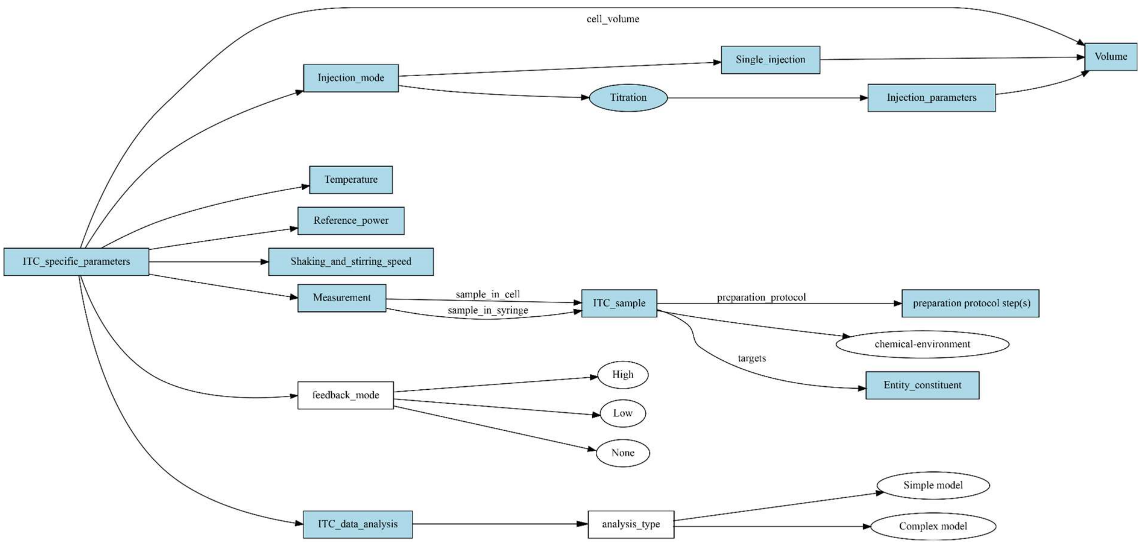

Fig. S6 Schema of the ITC-specific metadata under data blocks “Instrument”, “Measurements”, and “Data analysis” of the MBDB UI. For details see the full data model definitions.
